# The flavonoid compound apigenin prevents colonic inflammation and motor dysfunctions associated with high fat diet-induced obesity

**DOI:** 10.1371/journal.pone.0195502

**Published:** 2018-04-11

**Authors:** Daniela Gentile, Matteo Fornai, Rocchina Colucci, Carolina Pellegrini, Erika Tirotta, Laura Benvenuti, Cristina Segnani, Chiara Ippolito, Emiliano Duranti, Agostino Virdis, Sara Carpi, Paola Nieri, Zoltán H. Németh, Laura Pistelli, Nunzia Bernardini, Corrado Blandizzi, Luca Antonioli

**Affiliations:** 1 Department of Clinical and Experimental Medicine, University of Pisa, Pisa, Italy; 2 Department of Pharmaceutical and Pharmacological Science, University of Padova, Padova, Italy; 3 Department of Pharmacy, University of Pisa, Pisa, Italy; 4 Interdepartmental Research Center “Nutraceuticals and Food for Health”, University of Pisa, Pisa, Italy; 5 Department of Surgery, Morristown Medical Center, Morristown, New Jersey, United States of America; 6 Department of Agriculture, Food and Environment (DAFE), University of Pisa, Pisa, Italy; "INSERM", FRANCE

## Abstract

**Background and purpose:**

Apigenin can exert beneficial actions in the prevention of obesity. However, its putative action on obesity-associated bowel motor dysfunctions is unknown. This study examined the effects of apigenin on colonic inflammatory and motor abnormalities in a mouse model of diet-induced obesity.

**Experimental approach:**

Male C57BL/6J mice were fed with standard diet (SD) or high-fat diet (HFD). SD or HFD mice were treated with apigenin (10 mg/Kg/day). After 8 weeks, body and epididymal fat weight, as well as cholesterol, triglycerides and glucose levels were evaluated. Malondialdehyde (MDA), IL-1β and IL-6 levels, and *let-7f* expression were also examined. Colonic infiltration by eosinophils, as well as substance P (SP) and inducible nitric oxide synthase (iNOS) expressions were evaluated. Motor responses elicited under blockade of NOS and tachykininergic contractions were recorded *in vitro* from colonic longitudinal muscle preparations.

**Key results:**

When compared to SD mice, HFD animals displayed increased body weight, epididymal fat weight and metabolic indexes. HFD mice showed increments in colonic MDA, IL-1β and IL-6 levels, as well as a decrease in *let-7f* expression in both colonic and epididymal tissues. HFD mice displayed an increase in colonic eosinophil infiltration. Immunohistochemistry revealed an increase in SP and iNOS expression in myenteric ganglia of HFD mice. In preparations from HFD mice, electrically evoked contractions upon NOS blockade or mediated by tachykininergic stimulation were enhanced. In HFD mice, Apigenin counteracted the increase in body and epididymal fat weight, as well as the alterations of metabolic indexes. Apigenin reduced also MDA, IL-1β and IL-6 colonic levels as well as eosinophil infiltration, SP and iNOS expression, along with a normalization of electrically evoked tachykininergic and nitrergic contractions. In addition, apigenin normalized *let-7f* expression in epididymal fat tissues, but not in colonic specimens.

**Conclusions and implications:**

Apigenin prevents systemic metabolic alterations, counteracts enteric inflammation and normalizes colonic dysmotility associated with obesity.

## Introduction

Obesity represents one of the major health issues, with an alarmingly increasing prevalence around the world, leading to enormous social costs [1]. The International Obesity Taskforce has estimated that more than 300 million people are obese, and that the number of obese-born children in developing countries is increasing steadily [2].

Obesity is strongly linked to comorbidities, including gastrointestinal (GI) disorders that occur as gastroesophageal reflux disease, dyspepsia, constipation, irritable bowel syndrome, diarrhea, bloating and other non-specific conditions [3–5]. Preclinical studies, aimed at characterizing the molecular mechanisms underlying these GI disturbances, reported that diet-induced obesity determines a remarkable morpho-functional remodeling of the enteric neuromuscular compartment [6], followed by alterations of gut transit [7]. Several lines of evidence indicate the presence of an increased mucosal permeability, along with low grade enteric inflammation and oxidative stress in the bowel tissues of obese animals [8,9], leading to hypothesize a critical role of such phlogistic condition in the pathophysiology of intestinal dysfunctions associated with obesity [10].

Interestingly, several studies, aimed at finding out novel approaches for the management of obesity, have focused their attention on the potential beneficial effects of polyphenols, one of the most widely represented group of phytochemicals in the plant kingdom [11]. In recent years, several *in vitro* and *in vivo* studies showed that polyphenols might exert protective effect against oxidative stress-related diseases, including metabolic disorders, obesity and cancer [10].

The main class of polyphenols is represented by flavonoids, a large group of compounds characterized by a wide spectrum of biological actions, including antioxidant, free radical scavenger, metal ion chelating, vasoprotective, hepatoprotective, anti-inflammatory, anti-cancer, anti-infective and antidiabetes effects [2].

Among flavonoids, apigenin (4′,5,7,-trihydroxyflavone) has gained much attention as a beneficial and healthy compound owing to its wide spectrum of biological effects and low intrinsic toxicity. Indeed, this flavonoid can exert a remarkable anti-inflammatory activity [12], ameliorates obesity-related inflammation [13], and improves metabolic syndrome [14, 15]. Nevertheless, there is currently a lack of data about the putative beneficial effects resulting from apigenin supplementation on enteric functional disorders and inflammation related to obesity.

Based on the above background, the present study has been conceived to evaluate the effects of apigenin on colonic inflammatory and motor abnormalities in a mouse model of diet-induced obesity.

## Materials and methods

### Animals, diets and apigenin treatment

All experiments were approved by the Ethical Committee for Animal Experimentation at the University of Pisa and the Italian Ministry of Health (authorization n° 744/2015-PR). Animal care and handling were carried out in accordance with the directives of the European Community Council Directive 2010/63/UE, recognized and adopted by the Italian Government. Six week old male C57BL/6 mice (20–22 g body weight) were purchased from ENVIGO S.r.l (San Piero al Natisone, UD, Italy) and employed throughout the study. During the adaptation period (1 week), animals were housed in stainless-steel cages in a temperature-controlled (22–24°C) room, with a 12-h light/dark cycle and 50–60% relative humidity.

To induce obesity, mice were switched (at t = 0) from standard diet (SD, 18% kcal from fat; TD.2018), which was administered during the adaptation period to all mice, to a high-fat diet (HFD, 60% kcal from fat, TD.06414) for 8 weeks. HFD provided 18.3% kcal as proteins, 21.4% kcal as carbohydrates and 60.8% kcal as fat (5.1 kcal/g), whereas SD provided 24% kcal as proteins, 58% kcal as carbohydrates and 18% kcal as fat (3.1 kcal/g). Mice had free access to food and tap water ad libitum. After 1-week adaption under laboratory conditions, mice were randomly allocated to the following four experimental groups (n = 5 per group): 1) SD; 2) SD treated with apigenin (10 mg/Kg/day); 3) HFD; 4) HFD treated with apigenin (10 mg/Kg/day). Apigenin (purity ≥97% from parsley, Sigma Chemicals Co., St. Louis, MO, USA) was administered daily via oral gavage. The dose was selected in accordance with a previous report [16], and by preliminary experiments designed to assay increasing doses of apigenin (1, 10 and 30 mg/kg/day) on body weight, epididymal fat weight and colonic MDA levels in the model of HFD-induced obesity. Apigenin was dissolved in dimethyl sulfoxide (DMSO), and then diluted 1:1 with 0.5% carboxymethylcellulose (CMC, final volume 50 μl/mouse), a thickening agent commonly used as vehicle for oral gavage administrations. The administration of apigenin vehicle (1:1 DMSO:CMC) had been shown to not affect body weight gain, colonic MDA levels, blood glucose and cholesterol levels in preliminary experiments.

During the treatment body weight was measured once a week. At the end of the study, animals were fasted overnight, anaesthetized using chloral hydrate, and sacrificed by cervical dislocation. Blood samples (50 μl) were taken by tail incision and collected in tubes containing heparin to analyze systemic metabolic parameters. Colonic and white adipose tissue (epididymal fat) were removed, weighed and stored at -80°C until subsequent assays.

### Measurement of metabolic parameters

Total blood cholesterol, triglycerides and glucose levels were immediately assayed using MULTICARE IN (Biochemical Systems International S.r.l., Arezzo, Italy) in accordance with the manufacturer’s instructions.

### Evaluation of tissue malondialdehyde (MDA) levels

MDA concentration in colonic specimens was evaluated as described in detail previously by Antonioli and coworkers [17] in order to obtain a quantitative estimation of membrane lipid peroxidation. Colonic tissues were weighed, minced by forceps, homogenized in 2 ml of cold buffer (20 mM Ripa buffer, pH 7.4) by a polytron homogenizer (QIAGEN, Milan, Italy), and spun by centrifugation at 1600g for 10 min at 4°C. Colonic MDA levels were assessed with a kit for colorimetric assay (Calbiochem, San Diego, CA, U.S.A.), and the results obtained were expressed as nmol of MDA per milligram of colonic tissue.

### Evaluation of tissue IL-1β and IL-6 levels

IL-1β and IL-6 levels in colonic tissues were quantified by enzyme-linked immunosorbent assay (ELISA) kits (Abcam, Cambridge, UK), following the protocols provided by the manufacturer. Colonic tissue samples, stored previously at -80°C, were weighed, thawed, and homogenized in 0.4 ml of PBS, pH 7.2/20 mg of tissue at 4°C, and centrifuged at 10,000g for 5 min. Aliquots (100 μL) of supernatants were then used for the assay. IL-1β and IL-6 concentrations were expressed as picogram per milligram of tissue.

### Evaluation of tissue *let-7f* expression

Total microRNAs were purified and extracted from cryo-preserved colon (20–50 mg) and epididymal fat (100mg) by using miRNeasy Mini Kit (Qiagen, Germany). Reverse transcription of the extracted miRNAs was performed by the miScript Reverse Transcription Kit (Qiagen, Germany). cDNA was diluted 1:10 in RNase-free water and then qPCRs were performed in triplicate using the miScript SYBR-Green PCR kit (Qiagen, Germany) on the MiniOpticon CFX 48 real-time PCR Detection System (Bio-Rad, Hercules, USA). MiScript Primer Assays specific for mmu-*let-7f*-5p (MIMAT0000525) and the housekeeping mmu-RNU6 were obtained from Qiagen. *let-7f* expression was calculated using Ct method and normalized to the expression of RNU6.

### Histological analysis

8-μm-thick slices from full-thickness, formalin-fixed, paraffin-embedded colonic samples were processed to evaluate the morphology of colonic wall by haematoxylin/eosin and immunoperoxidase staining (see below). The severity and extent of inflammatory infiltrations were evaluated on the basis of the percentage of leucocytes per microscopic field and their presence through the colonic wall layers, respectively, as previously described [18]. The density of eosinophils was estimated within the *tunica mucosa/submucosa* and expressed as cell number per square millimeter as described in detail previously by Pellegrini and coworkers [19]. The microscopic analyses were performed by three different histologists (NB, CS, CI), blinded to the experimental group, using a Leica DMRB light microscope equipped with a computer image analysis software (L.A.S. software v.4.5), and following histomorphological scores for intestinal inflammation in mice [20].

### Immunohistochemistry of substance P (SP) and inducible nitric oxide synthase (iNOS)

Paraffin-sections were processed for immunoperoxidase staining, as described in detail previously by Ippolito and coworkers [21]. After an overnight incubation with primary antibodies against SP (code n. Sc-21715, Santa Cruz Biotech, California, USA) and iNOS (code n. ab 15323, Abcam, Cambridge, UK), the slices were exposed to appropriate biotinylated immunoglobulins, peroxidase-labelled streptavidin complex, and 3.3’-diaminobenzidine tetrahydrochloride (DakoCytomation, Glostrup, Denmark). The quantitative assessment of the immunostained sections was performed by the L.A.S. software v.4.5 Image Analysis System. The expression of antigens was evaluated as percentage of positive pixels (PPP) estimated over the total area of myenteric ganglia examined.

### Recording of colonic contractile activity

The contractile activity of colonic muscle preparations was recorded as reported in detail previously by Pellegrini et al. [19] with minor changes. Following sacrifice the colon was removed and placed into Krebs solution. Colonic specimens were cut along the longitudinal axis into strips of approximately 4-mm in width and 10-mm in length. The preparations were set up in organ baths containing Krebs solution at 37°C, bubbled with 95% O_2_ + 5% CO_2_, and connected to isometric force transducers (resting load = 0.5 g). Their mechanical activity was recorded by BIOPAC MP150 (Biomedica Mangoni, Pisa, Italy). Krebs solution had the following composition (mM): NaCl 113, KCl 4.7, CaCl_2_ 2.5, KH_2_PO_4_ 1.2, MgSO_4_ 1.2, NaHCO_3_ 25, glucose 11.5 (pH 7.4±0.1). Each colonic preparation was allowed to equilibrate for at least 30 min, with intervening washes at 10-min intervals. A pair of coaxial platinum electrodes was positioned at a distance of 10 mm from the longitudinal axis of each preparation to deliver electrical stimulations by a BM-ST6 stimulator (Biomedica Mangoni, Pisa, Italy). At the end of equilibration period, each preparation was repeatedly challenged with electrical stimuli, and the experiments started when reproducible motor responses were obtained (usually after two or three stimulations). Preliminary experiments were performed to select the appropriate electrical stimulation frequency or exogenous substance P concentrations. For this purpose, colonic preparations were challenged with single electrical stimuli at increasing frequencies, ranging from 1 to 20 Hz (10-s ES, 0.5 ms, 30 mA). Concentration–response curves to exogenous substance P were constructed at concentrations ranging from 0.01 to 10 μM in the presence of tetrodotoxin (TTX; 1 μM, Tocris, Bristol, UK). These preliminary experiments allowed to select the frequency of 10 Hz and the concentration of 1 μM for substance P (SP; Tocris, Bristol, UK).

In the first set of experiments, ES-induced contractions were recorded from colonic preparations maintained in standard Krebs solution.

In the second set of experiments, ES-induced contractions were assessed in colonic preparations maintained in Krebs solution containing guanethidine (10 μM, Sigma Chemicals Co., St. Louis, MO, USA), to prevent the recruitment of noradrenergic pathway. Under these conditions, ES-induced contractions were assessed in colonic preparations either in the absence and in the presence of N^ω^-nitro-L-arginine methylester (L-NAME; 100 μM, Sigma Chemicals Co., St. Louis, MO, USA), to prevent the recruitment of noradrenergic pathway.

In the third series, ES-evoked contractions were recorded form colonic preparations maintained in Krebs solution containing guanethidine, L-NAME, and atropine (1 μM, Sigma Chemicals Co., St. Louis, MO, USA), to prevent the recruitment of noradrenergic, nitrergic and cholinergic pathways. The NK_2_ receptor antagonist (GR159897, 1 μM, Tocris, Bristol, UK) and NK_3_ receptor antagonist (SB218795, 1 μM, Tocris, Bristol, UK) were added to examine the patterns of colonic excitatory motor responses mediated by the tachykininergic NK_1_ receptors pathway.

In the last series of experiments, tachykininergic contractions where evoked by direct pharmacological activation of tachykininergic NK_1_ receptors located on colonic smooth muscle cells. For this purpose, colonic preparations were maintained in Krebs solution containing TTX (1 μM) and stimulated with exogenous SP (1 μM).

### Statistical analysis

All data sets were represented as the mean ± standard error of mean (SEM). Comparisons of findings among groups were carried out by one-way analysis of variance (ANOVA). When interaction and/or the main effects were significant, means were compared using Newman-Keuls multiple-comparison *post-hoc* test. A *P* value<0.05 was considered as statistically significant. All statistical procedures were performed by commercial software (GraphPad Prism, version 3.0 from GraphPad Software Inc., San Diego, CA, USA).

## Results

### Body and epididymal fat weight

**Fig 1:**
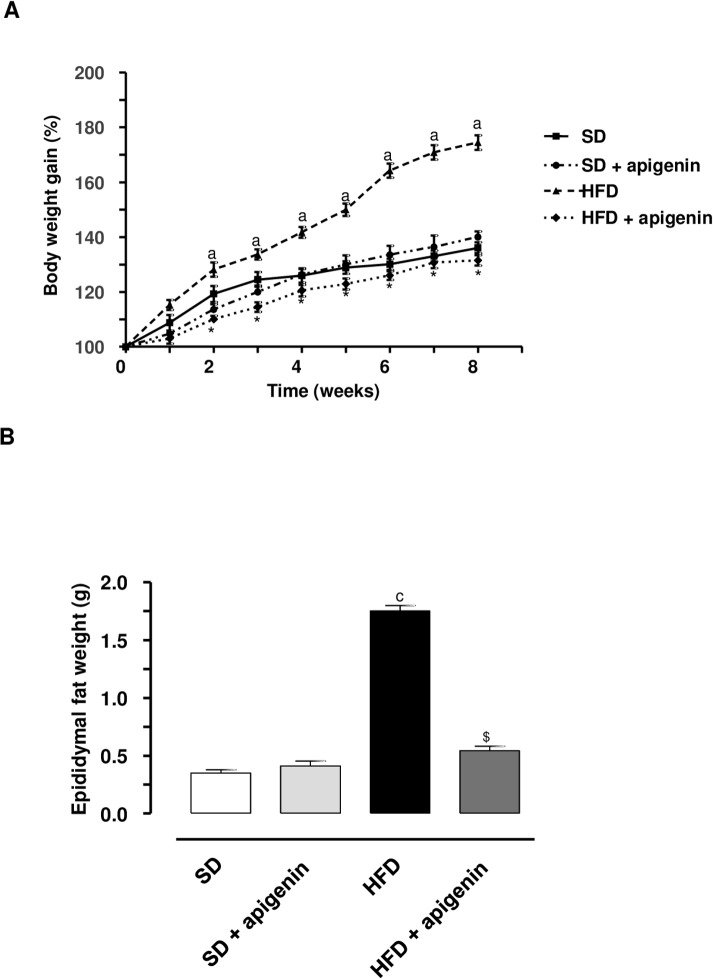
In HFD mice, apigenin counteracted the increase in body and epididymal fat weight. (A) Body weight gain (%) in mice fed with SD, SD plus treatment with apigenin (10 mg/Kg/day), HFD or HFD plus treatment with apigenin (10 mg/Kg/day) for 8 weeks. Values are means ± SEM, n = 5. ^a^*P*<0.05 significant difference vs SD at the respective weeks; *P<0.05 significant difference vs HFD at the respective weeks. (B) Epididymal fat weight in mice fed with SD, SD plus treatment with apigenin (10 mg/Kg/day), HFD or HFD plus treatment with apigenin (10 mg/Kg/day) for 8 weeks. Values are means±SEM, n = 5. ^c^*P*<0.001, significant difference vs SD; ^$^*P* <0.01 significant difference vs HFD.

HFD mice underwent a significant increase in body weight, as compared with SD animals (Fig 1A). Apigenin significantly counteracted the body weight gain in HFD mice (Fig 1A). In animals fed with SD, apigenin administration did not modify the pattern of body weight gain throughout the 8 weeks, as compared with SD animals (Fig 1A). The weight of epididymal fat did increase also in HFD animals, and apigenin counteracted significantly such an increment (Fig 1B). Epididymal fat weight did not differ significantly between SD and apigenin treated-SD mice (Fig 1B). No mice died and all remained in good clinical conditions throughout the experimental period.

### Measurements of metabolic parameters

The blood levels of metabolic parameters are displayed in Table 1. HFD resulted in a significant increase in all the assayed parameters, as compared with SD. Apigenin led to a significant reduction of total cholesterol, triglycerides and glucose levels in HFD mice, as compared to control HFD mice (Table 1). Moreover, there was no significant difference in metabolic parameter levels between SD group and apigenin treated-SD mice (Table 1).

**Table 1:** Apigenin reduced total cholesterol, triglycerides and glucose levels in HFD mice.

	SD	SD+Apigenin	HFD	HFD+Apigenin
Total cholesterol (mg/dL)	145±5.4	146±4.6	182±7.7^a^	142±5.3*
Triglycerides (mg/dL)	118±2.4	115±3.7	149.5±0.03^a^	109±6.8*
Glucose (mg/dL)	128±7	127±3	166±4.8^a^	110±7.3*

Blood metabolic parameters in SD, apigenin treated-SD mice (10 mg/Kg/day), HFD or apigenin treated-HFD mice (10 mg/Kg/day) for 8 weeks. Values are means±SEM, n = 5.

^a^*P* <0.05, significant difference vs SD

*P <0.05, significant difference vs HFD.

### Assessment of MDA, IL-1β and IL-6 levels in colonic tissues

**Fig 2:**
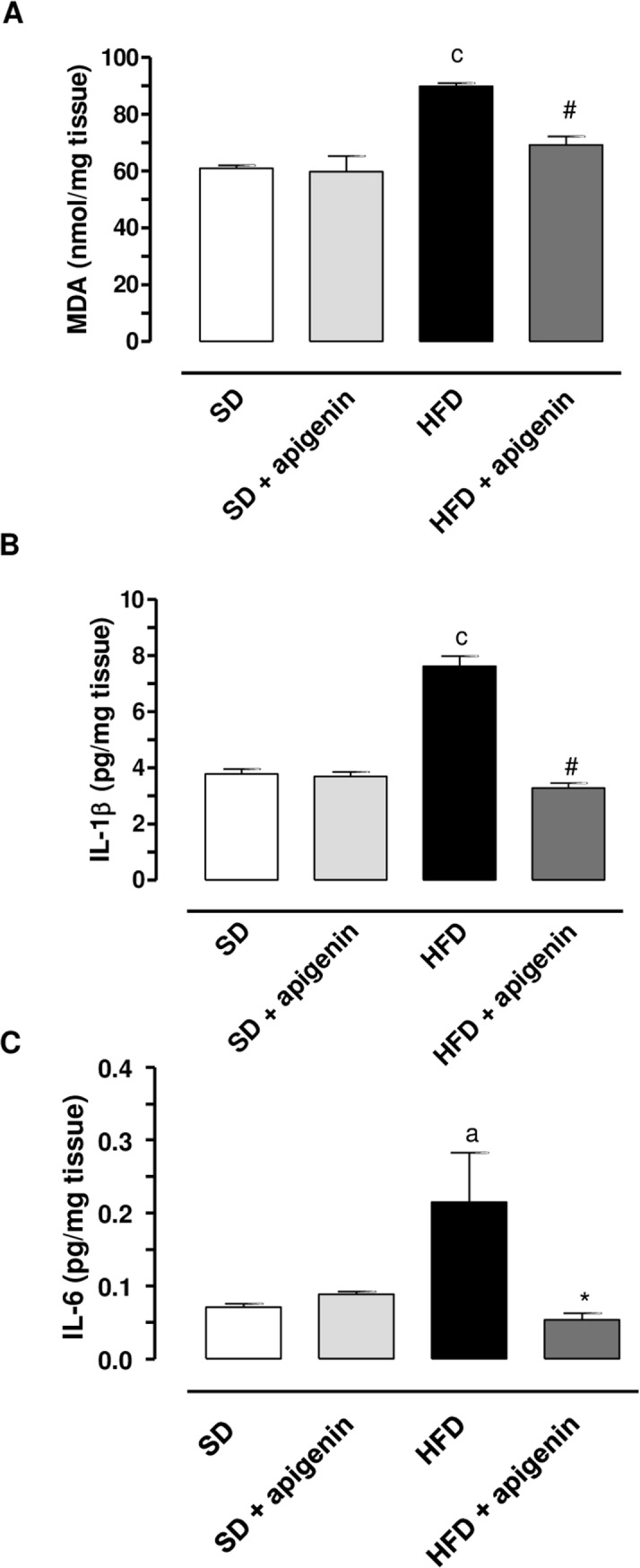
In HFD mice, apigenin decreased MDA, IL-1β and IL-6 colonic levels. MDA (A), IL-1β (B) and IL-6 (C) levels in colonic tissues from mice fed with SD, SD plus treatment with apigenin (10 mg/Kg/day), HFD or HFD plus treatment with apigenin (10 mg/Kg/day). Values are means±SEM, n = 5. ^a^*P* < 0.05, ^c^*P* < 0.001 significant difference vs SD; *P <0.05, ^#^*P*<0.001 difference difference vs HFD.

In colonic specimens from SD mice, MDA levels accounted for 61.6±1.6 nmol/mg (Fig 2A). HFD-induced obesity was associated with a significant increase in the oxidative stress (89.9±1.1 nmol/mg) (Fig 2A).

In colonic tissues from SD mice IL-1β and IL-6 levels were 3.6±0.04 pg/mg and 0.08±0.01 pg/mg, respectively (Fig 2B and 2C). In colonic specimens from obese mice, IL-1β and IL-6 levels were significantly increased as compared with SD mice (7.6±0.4 pg/mg and 0.22±0.5 pg/mg, respectively) (Fig 2B and 2C).

In SD mice apigenin did not affect MDA, IL-1β and IL-6 levels as compared with SD animals (Fig 2A, 2B and 2C). By contrast, the flavonoid determined a significant reduction of MDA, IL-1β and IL-6 levels in HFD mice, as compared to the levels found in untreated HFD mice (Fig 2A, 2B and 2C).

### Expression of *let*-7f in colonic and epididymal fat tissues

**Fig 3:**
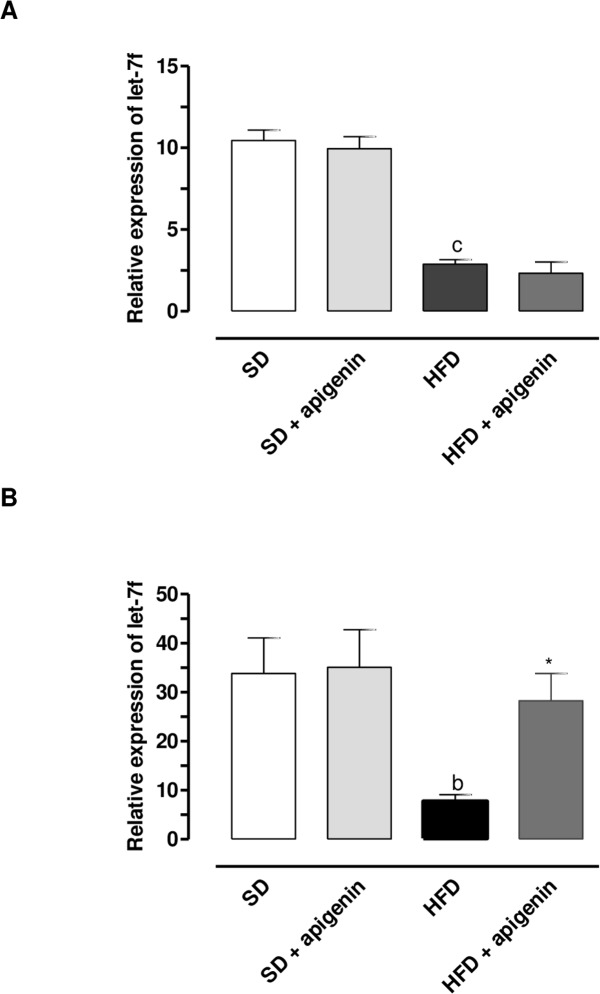
In HFD mice, apigenin normalized *let-7f* expression in epididymal fat tissues but not in colonic specimens. *let-7f* expression in colonic (A) and epididymal fat (B) tissues from SD, SD plus treatment with apigenin (10 mg/Kg/day), HFD or HFD plus treatment with apigenin (10 mg/Kg/day). Values are means±SEM, n = 5.^*c*^*P* < 0.001 significant difference vs SD, ^*b*^*P*<0.01 significant difference vs SD, ^*^P<0.05 significant difference vs HFD.

*let-7f* expression was significantly down-regulated in both colonic and epididymal fat tissues from HFD mice, as compared to the levels found in SD mice (Fig 3A and 3B). In SD mice, apigenin did not influence *let-7f* expression in both colonic and epididymal fat tissues (Fig 3A and 3B). In HFD mice, apigenin did not counteract the decreased expression of *let-7f* in colonic tissue (Fig 3A), while normalizing *let-7f* expression in epididymal fat tissue (Fig 3B).

### Histopathological analysis of eosinophil infiltration in colonic tissue

**Fig 4:**
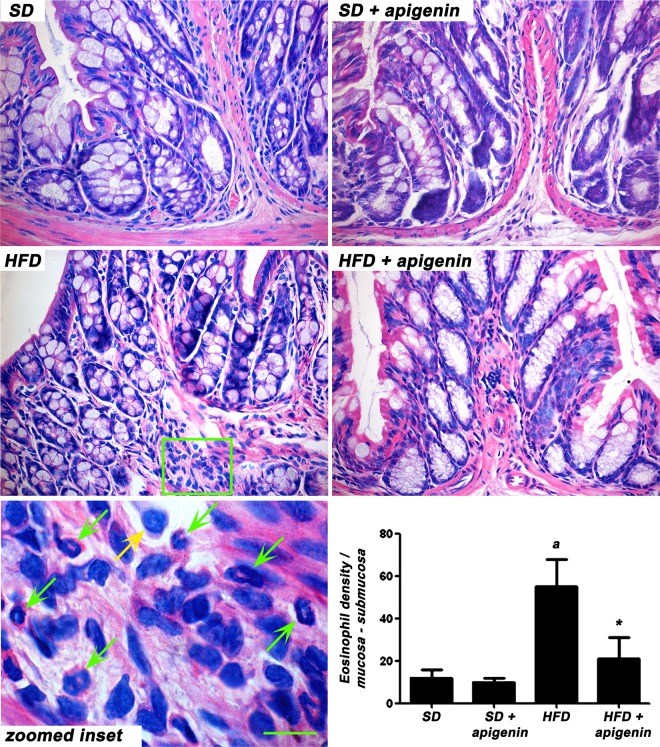
Apigenin decrease eosinophil infiltration in colonic tissues from HFD-mice. Representative microscopic pictures of haematoxylin/eosin-stained colonic sections from mice fed with SD, SD plus treatment with apigenin (10 mg/Kg/day), HFD or HFD plus treatment with apigenin (10 mg/Kg/day). The magnification within the boxed area shows the presence of eosinophils (green arrows) and neutrophils (yellow arrows). Scale bars = 50 μm; 20 μm (inset). The column graphs display the mean value of eosinophil density per square millimeter of *tunica mucosa/submucosa* areas (cell/mm^2^) ± SEM, n = 5. ^a^P<0.05 significant difference *vs* SD; *P<0.05 significant difference *vs* HFD.

Minimal (<10%) inflammatory infiltrates of mixed leucocytes were observed in the *tunica mucosa* and *submucosa* of colonic specimens from mice fed with HFD and HFD plus treatment with apigenin. The eosinophil infiltration, which reached the highest values in HFD group, was significantly counteracted by apigenin in HFD animals (Fig 4).

### Expression of SP and iNOS in colonic myenteric ganglia

**Fig 5:**
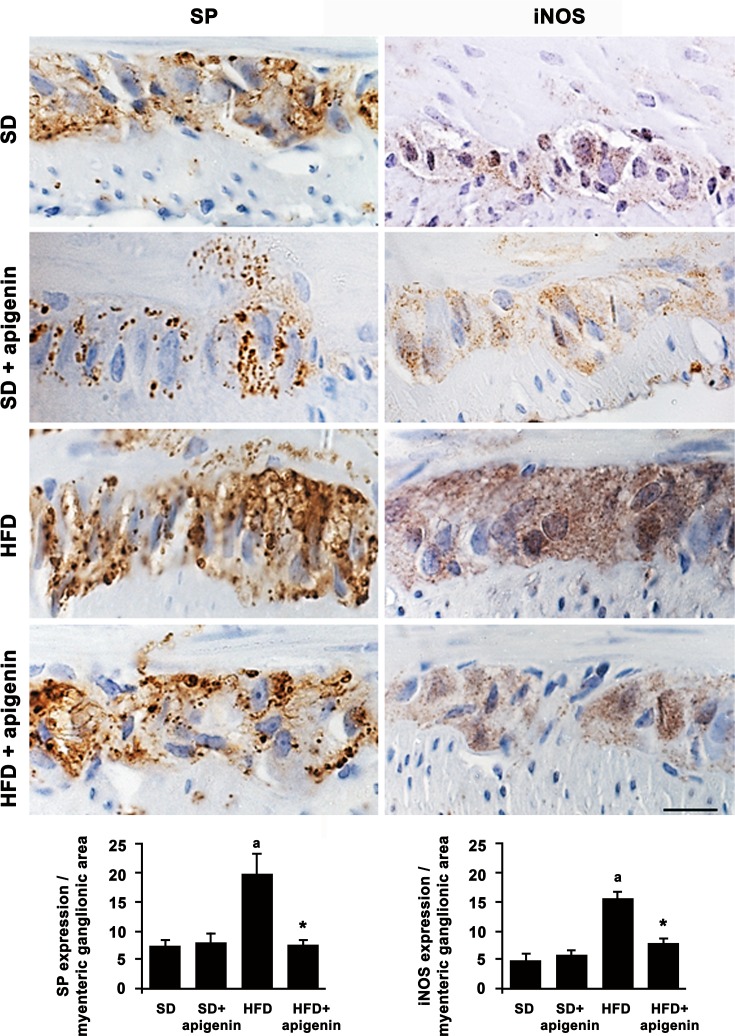
Apigenin reduced SP and iNOS expression in colonic myenteric ganglia from HFD mice. Representative pictures of SP and iNOS immunostaining of cross-sections from full-thickness mice colonic specimens from SD, SD plus treatment with apigenin (10 mg/Kg/day), HFD or HFD plus treatment with apigenin (10 mg/Kg/day). Scale bar = 20μm. Column graphs display the quantitative assessment of SP and iNOS in myenteric ganglion area. Each column represents the mean value of PPP±SEM, n = 5. ^a^*P*<0.05 significant difference vs SD; *P<0.05 significant difference vs HFD.

A significant increase in SP and iNOS immunostaining was found in colonic myenteric ganglia from HFD mice, as compared to SD animals (Fig 5). Apigenin did not affect the expression of both SP and iNOS in colonic myenteric ganglia of SD mice. However, the flavonoid prevented the increased expression of both markers in colonic myenteric ganglia induced by HFD (Fig 5).

### Motor activity of colonic longitudinal smooth muscle

During the equilibration period in standard Krebs solution, most colonic preparations displayed a rapid spontaneous motor activity, which remained stable throughout the experiment, in most cases, was low in amplitude, and did not interfere with motor responses evoked by ES. Electrically evoked responses consisted of phasic contractions followed in some cases, by after-contractions of variable amplitude.

**Fig 6:**
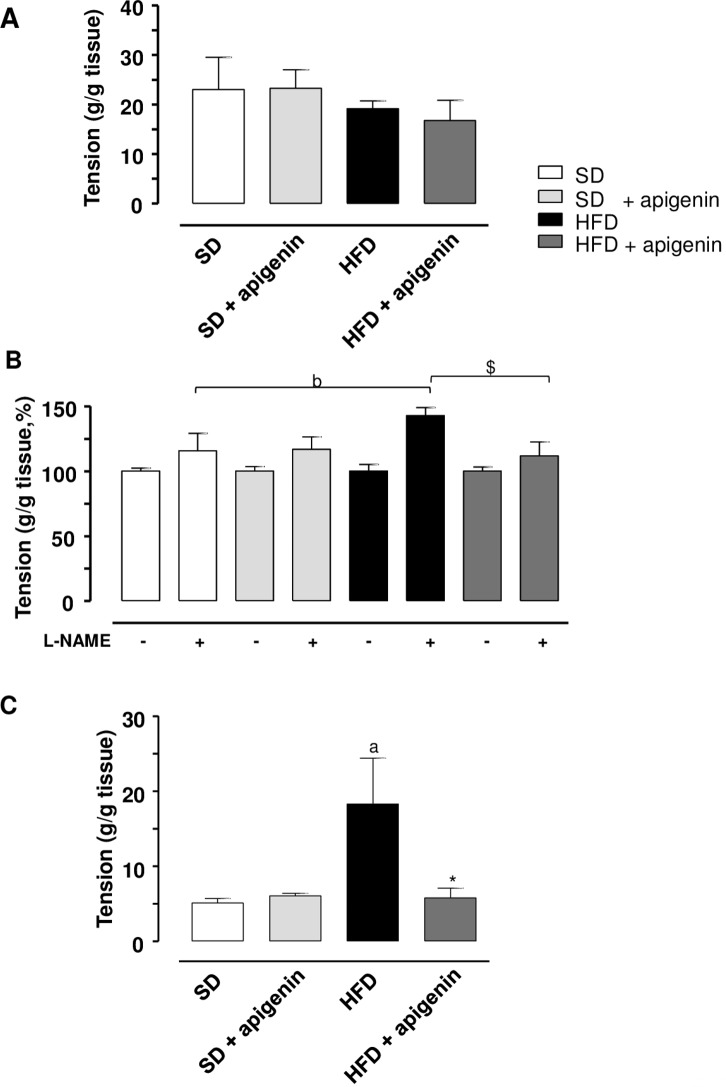
In HFD mice, apigenin normalized electrically evoked contractions elicited under NOS blockade or mediated by tachykininergic stimulation. Electrically evoked (ES, 10 Hz) contractile activity of colonic longitudinal smooth muscle preparations isolated from mice fed with SD, SD plus treatment with apigenin (10 mg/Kg/day), HFD, or HFD treated with apigenin (10 mg/Kg/day). (A) Colonic tissues maintained in standard Krebs solution. (B) Colonic tissues maintained in Krebs solution containing guanethidine (10 μM) and L-NAME (100 μM). (C) Colonic tissues maintained in Krebs solution containing guanethidine (10 μM), L-NAME (100 μM), GR159897 (1 μM), SB218795 (1 μM) and atropine (1 μM). Values are means±SEM, n = 5. ^a^*P*<0.05, ^b^*P*<0.01 significant difference vs SD; *P<0.05, ^$^*P*<0.01 significant difference vs HFD.

In colonic preparations from SD or HFD animals, maintained in standard Krebs solution, the application of ES elicited contractile responses, which accounted for 22.9±6.4g/g tissue and 19.2±1.5 g/g tissue, respectively (Fig 6A). Apigenin did not modify significantly the electrically evoked contractions in both SD and HFD mice (23.2±4 and 16.8±4.1 g/g tissue) (Fig 6A).

In colonic preparations from SD or HFD animals, incubated in Krebs solution added with guanethidine, electrically evoked contractions were similar to those recorded in preparations maintained in standard Krebs solution (20.3±3.4 g/g tissue and 18.2±1.2 g/g tissue, respectively). In this setting, apigenin did not significantly affect the electrically evoked contractions in both SD and HFD mice (21.4±2.3 and 17.2±2.1 g/g tissue).

In colonic preparations from HFD animals, maintained in Krebs solution containing guanethidine and L-NAME, the electrically evoked contractions recorded in the presence of NOS blockade were significantly enhanced (+48%) as compared with SD animals (Fig 6B). Apigenin counteracted the enhancement of electrically evoked contractions elicited under NOS blockade in HDF mice (Fig 6B), while it did not exert any significant effect in colonic tissues from SD fed mice (Fig 6B).

In colonic preparations, maintained in Krebs solution containing guanethidine, L-NAME, atropine, GR159897 and SB218795, the electrically evoked NK_1_-mediated tachykininergic contractions were significantly enhanced in HFD mice (+126%), as compared with SD mice (Fig 6C). In this setting, apigenin significantly reduced the enhanced contractions in HFD mice (Fig 6C), while it did not modify motor responses in colonic tissues from SD mice (Fig 6B). By contrast, the contractile responses evoked by incubation of colonic preparations with exogenous SP did not differ significantly in HFD and SD mice (25.6±2.3 and 42.8±2.7 g/g tissue, respectively). In this setting, apigenin did not affect SP-induced contractions in colonic preparations from HFD mice and SD mice (24.7±2.2 and 41.8±1.6 g/g tissue).

## Discussion

The present study was aimed at evaluating the effects of apigenin on colonic inflammatory and motor contractile abnormalities in a mouse model of diet-induced-obesity. The HFD-model of obesity is currently regarded as an important tool for understanding the impact of high-fat Western diets on the development of obesity and related disorders [22]. In our hands, consistently with previous reports [23–25], mice fed with HFD for 8 weeks displayed a marked increase in body weight and epididymal fat weight, associated with marked alterations of systemic metabolic indexes, such as blood total cholesterol, triglycerides and glucose levels, thus corroborating further the suitability of this experimental model. In addition, beyond the above mentioned alterations, HFD animals displayed a chronic low-grade systemic inflammation and immune system activation (e.g., a meta-inflammatory condition), characterized by a marked increase in pro-inflammatory cytokines (e.g., TNF, IL-1β and IL-6), which seem to be relevantly involved in the pathogenesis of obesity-related insulin resistance [26–29]. Of interest, in the present study signs of this mild inflammatory condition, which was associated with an increased level of oxidative stress, were observed in colonic specimens from HFD mice, as documented by the significant increment of IL-1β, IL-6 and MDA tissue concentrations and eosinophil density. These findings are in line with previous preclinical studies, reporting marked increments of IL-1β, IL-12p40 [30], IL-6, MDA and eosinophil infiltration [18] in colon tissues from HFD mice, as well as with clinical observations indicating an increase in IL-1β, IL-6, IL-8, TNF and monocyte chemotactic protein (MCP-1) in colonic mucosal samples from obese patients [31]. Our data on the expression of *let-7f* microRNA, which belongs to the highly conserved *let-7* family [32], are consistent with previous findings, showing a decrease in *let-7f* levels in the colonic mucosa of diet-induced obesity in mice, and an increase in *let-7f* levels in mice subjective to caloric restriction [33]. Moreover, *let-7f* reduced expression has been associated with an enhanced release of inflammatory chemokines in epithelial intestinal cells [34] and linked to the altered metabolic profile of obese patients [35, 36].

Over the years, a number of investigations has suggested that the presence of gut inflammation can undermine negatively the enteric motor functions, leading also to morphofunctional changes in the neuromuscular compartment [37]. In this regard, increasing epidemiologic data indicate that obesity is associated with chronic GI complaints [3], many of which overlap with common functional digestive disorders, such as gastroesophageal reflux, dyspepsia, constipation, irritable bowel syndrome, diarrhea, bloating and other non-specific conditions [38, 39]. These observations support the hypothesis of a tight link between the occurrence of inflammatory conditions at the enteric level and the onset of digestive motor abnormalities. In order to substantiate this relationship, as a first step, we evaluated, by means of immunohistochemical assays, the morphological rearrangements occurring in the colonic neuromuscular compartment of HFD mice. In particular, our morphological investigations allowed to observe that bowel tissues from obese animals displayed a significant increase in immunopositivity for iNOS and SP in myenteric ganglia, when compared with lean mice. Of note, these data are in accordance with our functional investigations, showing an increase in electrically evoked colonic contractions under NOS blockade, as well as in electrically tachykininergic contractions of colonic preparations from HFD mice.

A further set of experiments was dedicated to investigate whether the above mentioned magnification of colonic tachykininergic contractions could be ascribed to alterations occurring at muscular level in the presence of obesity. For this purpose, we tested the effect of a direct stimulation of NK_1_ receptors by exogenous SP in the presence of tetrodotoxin to abate neurogenic responses. Under these conditions, SP-induced contractions in colonic preparations from HFD animals did not differ from those recorded in SD mice. Overall, based on these findings, it is conceivable that this morphofunctional rearrangement of the colonic neuromuscular compartment in obese animals, characterized by an enhancement of both nitrergic and tachykininergic enteric neurotransmissions, could contribute significantly to the colonic motor abnormalities occurring in HFD mice. Indeed, a number of studies have widely described a critical involvement of the enteric nitrergic and tachykininergic pathways in the pathophysiology of digestive motor disorders associated with several inflammatory conditions [e.g., inflammatory bowel diseases (IBDs), diverticulitis and irritable bowel syndrome (IBS)] [40–43]. In particular, a marked increase in SP immunopositivity was observed in neurons of the myenteric and submucosal plexuses, as well as in immune cells of the intestinal *lamina propria* (such as monocytes, macrophages, eosinophils, and lymphocytes), isolated from patients with IBDs, leading to hypothesize an involvement of the enteric tachykininergic system in the pathogenesis of bowel motor dysfunctions associated with bowel inflammation [44]. In line with this view, a recent paper by Fornai et al. [42] reported the presence of bowel inflammation associated with enhancement of colonic excitatory tachykininergic motility in colonic tissues from patients with diverticular disease (DD), supporting the evidence of a pathogenic link between the occurrence of inflammation and a remodeling of enteric tachykininergic neurotransmission. In parallel, alterations of the enteric nitrergic pathway have been described also to be critically involved in enteric motor dysfunctions associated with inflammatory conditions [43]. In particular, upon induction of experimental colitis with dextran sulfate sodium (DSS), increased expression and activity of iNOS were observed, and such increments contributed to a delay in colonic transit [44–46].

Nowadays, an appropriate lifestyle and behavioral interventions are still a cornerstone in the management of obesity. However, maintaining such a healthy lifestyle is extremely challenging for patients. Therefore, nutraceutical research is paying increasing interest to the identification of novel compounds with beneficial effects on obesity and related comorbidities [2]. In this regard, several lines of evidence have shed light on the putative favorable effects of some natural compounds against obesity. In particular, flavonoids, polyphenol compounds abundantly contained in fruits and vegetables, seem to hold positive effects in this pathological setting, by virtue of their anti-inflammatory, antioxidant, and cardioprotective properties [2,47,48]. Among them, apigenin has revealed anti-obesity and anti-diabetic effects [49], due to its ability of suppressing the adipogenic process [50, 51] and regulating glucose tolerance [14].

Based on the above considerations, the second part of the present study was dedicated to evaluate the effects of apigenin in counteracting the colonic motor dysfunctions associated with HFD-induced obesity. Interestingly, in mice fed with HFD apigenin was effective in counteracting the increase in body and epididymal fat weight, as well as in reducing the elevations of blood total cholesterol, triglycerides and glucose. These findings corroborate previous observations highlighting an ameliorative effect of apigenin in obese mice in terms of glucose and lipid homeostasis, in parallel with the anorexigenic effects exerted by this flavonoid [14–16].

Of note, in our experiments, apigenin was effective also in mitigating the colonic inflammation and oxidative stress observed in HFD obese mice. An anti-inflammatory effect of apigenin was previously reported in a murine model of HFD-induced obesity, where its administration reduced plasma levels of MCP-1, TNF and IL-6 [52]. In addition, *in vitro* studies showed the efficacy of apigenin in attenuating the inflammation of adipose tissue, spurring the repolarization of infiltrating macrophages from an M1-proinflammatory phenotype toward an M2 anti-inflammatory population [13,53,54]. In line with these evidence, in our experiments, apigenin was also able to counteract the decreased expression of *let-7f* in epididymal fat tissue from HFD mice, likely due to its ability in regulating the inflammatory changes induced by HFD. By contrast, apigenin did not normalize *let-7f* expression in colonic tissue from HFD animals, suggesting that that the *let-7f* pathway does not play a relevant role in the anti-inflammatory effects exerted by apigenin at the enteric level.

Interestingly, apigenin, beyond its favorable effects on the systemic parameters altered by HFD, improved also the bowel morphofunctional abnormalities observed in obese mice. Indeed, our data showed that apigenin promoted a significant decrease in both iNOS and SP expression in myenteric ganglia, as well as a normalization of the electrically evoked contractions recorded under NOS blockade or mediated by tachykininergic receptors in HFD animals. Based on these findings, it is conceivable that apigenin, through a mitigation of chronic low-grade systemic and enteric inflammation, can counteract effectively the morphofunctional rearrangements of the colonic neuromuscular compartment occurring in the setting of obesity.

In conclusion, the present results indicate that apigenin can prevent metabolic alterations, such as the increase in body and epididymal fat weight as well as the elevation of blood total cholesterol, triglycerides and glucose levels, associated with HFD. Apigenin can counteract also the occurrence of colonic signs of mild inflammatory condition (IL-1β, IL-6 and MDA elevations) induced by HFD. Moreover, our data provide evidence, for the first time, that apigenin, under a condition of obesity, can normalize bowel dysmotility thought morphofunctional rearrangements, characterized by an enhancement of both nitrergic and tachykininergic enteric neurotransmissions. These experimental findings should stimulate further research for a better elucidation of the molecular mechanisms underlying the beneficial effects of this flavonoid, as well as clinical investigations aimed at assessing the efficacy of apigenin diet supplementation for the management of obesity and related inflammatory/functional bowel disorders.
